# *phi* and *phi*D predict adverse pathological features after radical prostatectomy for prostate cancer in Chinese population

**DOI:** 10.1002/cam4.70085

**Published:** 2024-08-09

**Authors:** Ruofan Shi, Da Huang, Jiaqi Yan, Xiaohao Ruan, Jingyi Huang, Jiacheng Liu, Jinlun Huang, Yongle Zhan, Chi Yao, Tsun Tsun Stacia Chun, Brian Sze‐Ho Ho, Ada Tsui‐Lin Ng, Yi Gao, Danfeng Xu, Rong Na

**Affiliations:** ^1^ Department of Urology Ruijin Hospital, Shanghai Jiao Tong University School of Medicine Shanghai China; ^2^ Division of Urology, Department of Surgery School of Clinical Medicine, LKS School of Medicine, The University of Hong Kong Hong Kong China; ^3^ Division of Urology, Department of Surgery Queen Mary Hospital Hong Kong China

**Keywords:** pathological outcomes, *phi* density, prostate cancer, Prostate Health Index, radical prostatectomy

## Abstract

**Background:**

Anticipating the postoperative pathological stage and potential for adverse features of prostate cancer (PCa) patients before radical prostatectomy (RP) is crucial for guiding perioperative treatment.

**Methods:**

A cohort consisting of three sub‐cohorts with a total of 709 patients has been enlisted from two major tertiary medical centres in China. The primary assessment parameters for adverse pathological features in this study are the pathological T stage, the AJCC prognostic stage groups and perineural invasion (PNI). Logistic regression analyses were performed to investigate the relationship between prostate specific antigen (PSA), its derivatives (incluing Prostate Health Index, *phi* and *phi* density, *phi*D), and the pathological outcomes after RP.

**Results:**

Both *phi* and *phi*D showed a significant association with pathologic T stage of pT3 or above (*phi*, adjusted OR, AOR = 2.82, 95% confidence interval, 95% CI: 1.88–4.23, *p* < 0.001; *phi*D, AOR = 2.47, 95% CI: 1.76–3.48, *p* < 0.001) and PNI (*phi*, AOR = 2.15, 95% CI: 1.39–3.32, *p* < 0.001; *phi*D, AOR = 1.94, 95% CI: 1.38–2.73, *p* < 0.001). In a subgroup analysis with a total PSA value <10 ng/mL, *phi* and *phi*D continued to show a significant correlation with pT3 or above (*phi*, AOR = 4.70, 95% CI: 1.29–17.12, *p* = 0.019; *phi*D, AOR = 3.44, 95% CI: 1.51–7.85, *p* = 0.003), and *phi*D also maintained its predictive capability for PNI in this subgroup (AOR = 2.10, 95% CI: 1.17–3.80, *p* = 0.014). Sensitivity analysis indicated that the findings in the combined cohort are mainly influenced by one of the sub‐cohorts, partially attributable to disparities in sample sizes between sub‐cohorts. Combined analysis of *phi*(D) and multiparametric MRI (mpMRI) data yielded similar results.

**Conclusions:**

Preoperative measurement of serum *phi* and *phi*D is valuable for predicting the occurrence of adverse pathological features in Chinese PCa patients after RP.

## 1 INTRODUCTION

Prostate cancer (PCa) has become the second most common cancer among men worldwide nowadays.^1^ According to the most recent cancer statistics (GLOBOCAN 2020), new cases and deaths in China account for 8.2% and 13.6% of the global PCa incidence and mortalities every year.^2^ As one of the most critical health challenges, extensive efforts have been dedicated to advancing the diagnosis, treatment and prognostic prediction of PCa. Radical prostatectomy (RP), as an effective treatment modality for localized PCa, offers valuable insights through its postoperative pathological findings, which can provide crucial guidance for prognosis and inform subsequent treatment decisions.

In *The Eighth Edition of the AJCC Cancer Staging Manual*, pathological T3 stage (pT3) is subdivided into pT3a and pT3b, where pT3a includes extracapsular extension (ECE), and pT3b indicates seminal vesicle invasion (SVI) of PCa. ECE and SVI, as well as positive surgical margins, are all considered adverse pathological features following RP. According to the latest National Comprehensive Cancer Network (NCCN) guidelines,^3^ in the risk stratification of PCa, the T3a stage is categorized as high‐risk, while T3b or above stages are classified into the very high‐risk group. Studies have previously established a correlation between pT3 and adverse prognosis in PCa,^4^, ^5^ and it is recommended to administer postoperative adjuvant radiotherapy^6^ and androgen deprivation therapy^7^ in pT3 PCa patients. Therefore, the ability to predict a patient's potential for adverse pathological features as early as at the initial visit, holds significant value in guiding the need for radical treatment along with adjuvant therapies.

So far, a number of studies have been conducted to predict the pathological outcomes before RP. In earlier studies,^8^, ^9^ researchers focused on combining the predictive role of serum prostate specific antigen (PSA) level, preoperative clinical stage and biopsy Gleason score (GS) in preoperative pathological staging for patients with clinically localized PCa. This led to the development of the Partin Tables,^10^ which reveal their efficiency in indicating whether the tumour is organ‐confined, has lymph node involvement, or shows ECE or SVI, but they do not precisely correlate with pathological T stages.

With the progression of biomarkers in PCa research, significant milestones were achieved with the FDA's approval of free PSA (fPSA) and [−2]proPSA (p2PSA), along with the Prostate Health Index (*phi*).^11^ As these approvals have marked significant advancements in the diagnostic approach for PCa, they have also spurred further research into predicting post‐RP pathological outcomes in PCa patients.^12^, ^13^, ^14^, ^15^ Many of these studies predominantly focused on foreign populations, with only a handful addressing specific regions in China. These latter studies often grappled with issues like limited sample sizes or inconsistent specimen resources. This scenario has motivated a deeper investigation into the relationship between PCa biomarkers and the occurrence of post‐RP adverse pathological features. Towards this goal, in our current study, we included over 700 subjects from two research centres in China. By combining preoperative blood tests and imaging data with postoperative pathological results, we aimed to investigate the predictive capacity of preoperative serum PSA and its derivatives upon occurrence of adverse pathological features in specimens after RP in Chinese PCa patients.

## 2 SUBJECTS AND METHODS

### 2.1 Study population

A total number of 709 prostate cancer (PCa) patients were included in the present study from two tertiary medical centres (Ruijin Hospital, Shanghai, and Queen Mary Hospital, Hong Kong) in China. The study subjects were derived from three different study sub‐cohorts: (sub‐cohort 1) a prospective biopsy cohort from Ruijin Hospital which has been described elsewhere^16^; (sub‐cohort 2) an RP cohort at Queen Mary Hospital in Hong Kong from 2000 to 2020; (sub‐cohort 3) another RP cohort at Queen Mary Hospital in Hong Kong from 2003 to 2022 with preoperative multiparametric MRI (mpMRI) data. Patients were included in the present analysis if: (1) pathologically diagnosed through ultrasound‐guided biopsies and then underwent open or laparoscopic or robotic‐assisted radical prostatectomy (RP); (2) with post‐operation pathological diagnosis including Gleason score (GS) or Gleason grade group, the extent of tumour infiltration, such as pathologic T stage and perineural invasion (PNI), and regional lymph node metastasis; (3) with pre‐biopsy total prostate specific antigen (tPSA), free PSA (fPSA) and prostate health index(*phi*) test results. Informed consent was obtained from each participant, and the study was approved by the institutional review board (IRB) of the ethics in each medical centre.

### 2.2 Clinical characteristics and pathological assessment parameters

The baseline clinical characteristics of patients registered in this study include age, prostate volume (PV) measured by ultrasound and estimated using the prostate ellipsoid formula ($=\left[\pi /6\right]\times \text{length}\times \text{width}\times \text{height}$), the number of biopsy‐positive cores (as a binary categorical variable, ≥3 cores as positive). tPSA and its derivatives include fPSA, PSA density (PSAD, =tPSA/PV), [−2]proPSA(p2PSA), p2PSA density (p2PSAD, =p2PSA/PV), *phi* ($=\left(p2\mathrm{PSA}/\text{fPSA}\right)\times \sqrt{\text{tPSA}}$) and *phi* density (*phi*D, =phi/PV). For sub‐cohort 3, preoperative PI‐RADS category and whether mpMRI suggested extracapsular extension (ECE) or seminal vesicle invasion (SVI) were also recorded (as a binary categorical variable, either ECE or SVI existence was regarded as mpMRI positive).

Primary assessment parameters of adverse pathological features in this study include the pathologic T stage (pT3 or above [abbreviated below as pT3+] and pT3b) and AJCC prognostic stage groups of PCa (stage group III or above [abbreviated below as Stage3+]), which are both based on *The Eighth Edition of the AJCC Cancer Staging Manual*, and PNI of PCa as well (according to the postoperative pathology reports given by the pathology department of each hospital).

### 2.3 Statistical and meta‐analysis

The comparison of baseline clinical characteristics across sub‐cohort 1 and 2 was carried out by Mann–Whitney *U* test (for continuous variables), Fisher's exact test (for categorical variables), and Cuzick's test for trend (for ordered categorical variables). The baseline clinical data for sub‐cohort 3 were presented separately due to its distinct data content and the different use for which they were analysed. To discover the underlying association between potential predictors and post‐RP adverse pathological outcomes, we applied univariable and multivariable logistic regression (LR) and calculated the odds ratio (OR) and adjusted OR (AOR), 95% confidence interval (95% CI), and the corresponding *p* value. In the multivariable LR, tPSA, p2PSA and *phi* have been adjusted for age, PV and number of positive cores (as a binary categorical variable, >2 vs. ≤2) after logarithmic transformation, while PSAD, p2PSAD and *phi*D have been adjusted for age and number of positive cores after logarithmic transformation. mpMRI results have also been adjusted for age and number of positive cores in the multivariable LR. Meta‐analytical methods include inverse variance method, *Q* test and $I^{2}$ for heterogeneity assessment and quantification, and leave‐one‐out method for sensitivity analysis. Furthermore, we employed fixed‐effect models for each of our analyses given the identified heterogeneity.

All statistical analyses and meta‐analysis were performed using R version 4.3.2.^17^ A two‐tailed *p* < 0.05 was considered statistically significant.

## 3 RESULTS

The baseline clinical characteristics of the patients from the first two sub‐cohorts are described in Table 1, with the corresponding AJCC prognostic stage groups distribution noted according to criteria listed in Table S1. In the combined cohort (Table 1), 45.9% of patients were defined as AJCC prognostic stage group III or above (Stage3+), while 34.2% of patients had a pathologic T stage pT3 or above (pT3+). Statistical significance between the two sub‐cohorts suggested the heterogeneity of the baseline characteristics. Specifically, there were significant differences between the two sub‐cohorts in tPSA and its derivatives (all *p* < 0.001), Gleason Grade Group (*p* for trend <0.001), perineural invasion (PNI, *p* < 0.001), pathologic and prognostic staging (*p* for trend <0.001).

**TABLE 1:** Baseline clinical characteristics of sub‐cohort 1 and 2.

Characteristics^a^	Combined Cohort (*N* = 348)	Sub‐cohort 1 (*n* = 225)	Sub‐cohort 2 (*n* = 123)	*P* ^b^
Age (years), median (IQR)	69 (64–73)	69 (64–74)	68 (64–71)	0.029
Prostate volume (mL), median (IQR)	34.3 (25.1–48.1)	32.3 (24.6–45.8)	36.1 (27.1–51.9)	0.051
Number of positive cores				
≥ 3, *n* (%)	184 (61.1)	143 (63.6)	41 (53.9)	0.173
≤ 3, *n* (%)	117 (38.9)	82 (36.4)	35 (46.1)	
Total PSA (ng/mL), median (IQR)	11.0 (7.5–19.4)	15.0 (9.2–27.2)	7.8 (6.2–11.0)	<0.001
PSAD (ng/mL^2^), median (IQR)	0.3 (0.2–0.6)	0.5 (0.3–1.0)	0.2 (0.1–0.3)	<0.001
p2PSA (pg/mL), median (IQR)	22.4 (13.7–39.3)	27.5 (15.2–52.7)	16.9 (12.0–25.3)	<0.001
p2PSAD (pg/mL^2^), median (IQR)	0.6 (0.4–1.3)	0.8 (0.4–1.7)	0.5 (0.3–0.6)	<0.001
*phi*, median (IQR)	52.7 (35.8–84.7)	61.7 (35.3–117.0)	45.3 (35.8–57.7)	<0.001
*phi*D, median (IQR)	1.6 (0.9–3.0)	1.8 (0.9–3.8)	1.2 (0.8–2.0)	<0.001
RP GGG, *n* (%)				
1 (≤6)	54 (15.5)	19 (8.4)	35 (28.5)	*p* for trend <0.001
2 (3 + 4)	157 (45.1)	107 (47.6)	50 (40.7)	
3 (4 + 3)	75 (21.6)	51 (22.7)	24 (19.5)	
4 (8)	22 (6.3)	14 (6.2)	8 (6.5)	
5 (9–10)	40 (11.5)	34 (15.1)	6 (4.9)	
RP GS ≥ 7, *n* (%)	294 (84.5)	206 (91.6)	88 (71.5)	<0.001
RP GG ≥ 3, *n* (%)	137 (39.4)	99 (44.0)	38 (30.9)	0.022
RP GS ≥ 8, *n* (%)	62 (17.8)	48 (21.3)	14 (11.4)	0.027
Perineural invasion, *n* (%)	232 (67.6)	167 (75.9)	65 (52.8)	<0.001
Positive surgical margin, *n* (%)	134 (38.6)	90 (40.0)	44 (36.1)	0.491
Pelvic lymph node metastases, *n* (%)	4/103 (3.9)	3/67 (4.5)	1/36 (2.8)	1.000
Pathologic T stage, *n* (%)				
pT2	229 (65.8)	130 (57.8)	99 (80.5)	*p* for trend <0.001
pT3a	70 (20.1)	53 (23.6)	17 (13.8)	
pT3b	48 (13.8)	41 (18.2)	7 (5.7)	
pT4	1 (0.3)	1 (0.4)	0 (0.0)	
AJCC prognostic stage groups, *n* (%)				
Stage I	32 (9.2)	8 (3.6)	24 (19.5)	*p* for trend <0.001
Stage IIA	16 (4.6)	6 (2.7)	10 (8.1)	
Stage IIB	102 (29.3)	61 (27.1)	41 (33.3)	
Stage IIC	38 (10.9)	18 (8.0)	20 (16.3)	
Stage IIIA	30 (8.6)	29 (12.9)	1 (0.8)	
Stage IIIB	88 (25.3)	67 (29.8)	21 (17.1)	
Stage IIIC	38 (10.9)	33 (14.7)	5 (4.1)	
Stage IVA	4 (1.1)	3 (1.3)	1 (0.8)	
Stage IVB	0 (0.0)	0 (0.0)	0 (0.0)	

^a^ GGG, Gleason grade group; GS, Gleason score; PSA, prostate specific antigen; PSAD, PSA density; p2PSA, [−2]proPSA; p2PSAD, p2PSA density; *phi*, prostate health index; *phi*D, *phi* density; RP, radical prostatectomy.

^b^ *p* for trend is calculated through Cuzick's test for trend, and a two‐tailed *p* < 0.05 was considered statistically significant.

Univariable and multivariable logistic regression (LR) were then performed to evaluate the association between various clinical predictors and adverse pathological features after radical prostatectomy (RP). The results, as shown in Table 2, revealed that, except for age and prostate volume (PV), all the other clinical characteristics were significant predictors of adverse pathological features in terms of pT3+, or pT3b specifically, Stage3+, or PNI in RP specimens (*p* < 0.001). In the multivariable LR analysis after adjusting for corresponding covariates, similar results were observed. Among these predictors, tPSA was significantly correlated with pT3b (adjusted odds ratio, AOR = 2.48, 95% confidence interval, 95% CI: 1.63–3.79, *p* < 0.001) and Stage3+ (AOR = 5.99, 95% CI: 3.57–10.07, *p* < 0.001). On the other hand, *phi* and *phi*D exhibited a strong association with pT3+ (*phi*, AOR = 2.82, 95% CI: 1.88–4.23, *p* < 0.001; *phi*D, AOR = 2.47, 95% CI: 1.76–3.48, *p* < 0.001) and PNI (*phi*, AOR = 2.15, 95% CI: 1.39–3.32, *p* < 0.001; *phi*D, AOR = 1.94, 95% CI: 1.38–2.73, *p* < 0.001).

**TABLE 2:** Logistic regression analysis for prediction of adverse pathological features after radical prostatectomy.

Predictor	pT3 or above (pT3+)				pT3b			
	Univariable LR analysis		Multivariable LR analysis		Univariable LR analysis		Multivariable LR analysis	
	OR (95% CI)	*p*	AOR (95% CI)	*p*	OR (95% CI)	*p*	AOR (95% CI)	*p*
Age	1.01 (0.98–1.05)	0.492	1.02 (0.98–1.06)	0.425	1.00 (0.95–1.04)	0.85	0.98 (0.93–1.04)	0.565
Prostate volume	0.98 (0.97–0.99)	0.003*	0.98 (0.97–1.00)	0.018*	0.98 (0.97–1.00)	0.108	0.99 (0.97–1.01)	0.469
# of positive cores ≥ 3	3.30 (1.95–5.60)	<0.001*	2.28 (1.26–4.13)	0.007*	18.07 (4.29–76.13)	<0.001*	13.96 (3.10–62.86)	<0.001*
Total PSA	2.41 (1.76–3.29)	<0.001*	2.04 (1.45–2.87)	<0.001*	2.88 (1.98–4.20)	<0.001*	2.48 (1.63–3.79)	<0.001*
PSAD	2.47 (1.84–3.33)	<0.001*	2.03 (1.48–2.77)	<0.001*	2.62 (1.84–3.74)	<0.001*	2.20 (1.49–3.27)	<0.001*
p2PSA	2.07 (1.58–2.73)	<0.001*	2.04 (1.51–2.77)	<0.001*	1.92 (1.41–2.61)	<0.001*	1.78 (1.26–2.52)	0.001*
p2PSAD	2.39 (1.81–3.17)	<0.001*	2.07 (1.55–2.77)	<0.001*	2.01 (1.48–2.74)	<0.001*	1.73 (1.23–2.42)	0.002*
*phi*	3.69 (2.50–5.45)	<0.001*	2.82 (1.88–4.23)	<0.001*	3.06 (1.97–4.77)	<0.001*	2.28 (1.42–3.67)	<0.001*
*phi*D	3.03 (2.17–4.23)	<0.001*	2.47 (1.76–3.48)	<0.001*	2.51 (1.69–3.73)	<0.001*	1.90 (1.25–2.89)	0.003*

Predictor	AJCC Stage III or above (Stage3+)				Perineural invasion (PNI)			
	Univariable LR analysis		Multivariable LR analysis		Univariable LR analysis		Multivariable LR analysis	
	OR (95% CI)	*p*	AOR (95% CI)	*p*	OR (95% CI)	*p*	AOR (95% CI)	*p*
Age	1.03 (0.99–1.06)	0.125	1.04 (0.99–1.08)	0.125	1.01 (0.98–1.05)	0.544	1.01 (0.96–1.05)	0.816
Prostate volume	0.99 (0.98–1.00)	0.054	0.99 (0.97–1.00)	0.069	0.99 (0.98–1.00)	0.045*	0.99 (0.98–1.00)	0.077
# of positive cores ≥ 3	3.06 (1.89–4.97)	<0.001*	1.75 (0.96–3.17)	0.066	4.85 (2.86–8.24)	<0.001*	4.01 (2.19–7.32)	<0.001*
Total PSA	6.40 (4.08–10.03)	<0.001*	5.99 (3.57–10.07)	<0.001*	2.44 (1.68–3.54)	<0.001*	1.95 (1.28–2.98)	0.002*
PSAD	4.85 (3.26–7.23)	<0.001*	4.06 (2.67–6.18)	<0.001*	2.26 (1.62–3.16)	<0.001*	1.96 (1.35–2.84)	<0.001*
p2PSA	3.79 (2.65–5.41)	<0.001*	4.43 (2.86–6.86)	<0.001*	1.67 (1.24–2.23)	<0.001*	1.44 (1.03–2.02)	0.033*
p2PSAD	3.90 (2.75–5.53)	<0.001*	3.62 (2.48–5.30)	<0.001*	1.82 (1.36–2.43)	<0.001*	1.54 (1.12–2.11)	0.008*
*phi*	4.49 (2.97–6.81)	<0.001*	3.72 (2.37–5.83)	<0.001*	2.86 (1.91–4.26)	<0.001*	2.15 (1.39–3.32)	<0.001*
*phi*D	2.89 (2.11–3.95)	<0.001*	2.53 (1.81–3.54)	<0.001*	2.21 (1.63–2.99)	<0.001*	1.94 (1.38–2.73)	<0.001*

Abbreviations: AOR, adjusted odds ratio; CI, confidence interval; LR, logistic regression; OR, odds ratio.

A two‐tailed *p* value < 0.05 was considered statistically significant and is indicated by an asterisk (*).

Given that the *phi* test is not conventionally performed in patients with a tPSA value ≥10 ng/mL in the sub‐cohort 2, a subgroup analysis (Table 3) was performed among patients with a preoperative tPSA value <10 ng/mL (*n* = 150). After eliminating the patients with a preoperative tPSA value ≥10 ng/mL, tPSA and its derivatives were no longer significantly associated with pT3b. However, p2PSA and p2PSAD remained closely associated with Stage3+ (p2PSA, AOR = 3.23, 95% CI: 1.27–8.18, *p* = 0.014; p2PSAD, AOR = 3.53, 95% CI: 1.47–8.47, *p* = 0.005). In addition, *phi* and *phi*D had also retained their correlation with pT3+ (*phi*, AOR = 4.70, 95% CI: 1.29–17.12, *p* = 0.019; *phi*D, AOR = 3.44, 95% CI: 1.51–7.85, *p* = 0.003). Besides, *phi*D was also found to be capable of predicting PNI after RP (AOR = 2.10, 95% CI: 1.17–3.80, *p* = 0.014).

**TABLE 3:** tPSA <10 ng/mL subgroup logistic regression analysis results (*n* = 150).

Predictor	pT3 or above (pT3+)				pT3b			
	Univariable LR analysis		Multivariable LR analysis		Univariable LR analysis		Multivariable LR analysis	
	OR (95% CI)	*p*	AOR (95% CI)	*p*	OR (95% CI)	*p*	AOR (95% CI)	*p*
Age	1.05 (0.97–1.13)	0.256	1.08 (0.99–1.19)	0.098	0.98 (0.85–1.13)	0.774	0.92 (0.76–1.12)	0.394
Prostate volume	0.96 (0.93–1.00)	0.027*	0.97 (0.93–1.00)	0.062	0.95 (0.89–1.02)	0.175	0.99 (0.91–1.07)	0.746
# of positive cores ≥ 3	2.05 (0.83–5.07)	0.121	1.70 (0.60–4.79)	0.317	N/A		N/A	
Total PSA	1.84 (0.45–7.57)	0.399	1.85 (0.39–8.76)	0.438	3.55 (0.15–84.15)	0.433	3.03 (0.04–222.87)	0.613
PSAD	3.65 (1.41–9.44)	0.008*	2.75 (0.98–7.71)	0.054	3.67 (0.72–18.75)	0.118	1.57 (0.19–12.97)	0.674
p2PSA	1.77 (0.93–3.37)	0.083	3.23 (1.27–8.18)	0.014*	1.14 (0.34–3.84)	0.832	1.47 (0.26–8.42)	0.665
p2PSAD	3.47 (1.65–7.29)	0.001*	3.53 (1.47–8.47)	0.005*	1.78 (0.62–5.13)	0.283	1.31 (0.26–6.66)	0.741
*phi*	5.11 (1.80–14.50)	0.002*	4.70 (1.29–17.12)	0.019*	4.09 (0.92–18.27)	0.065	2.10 (0.20–22.11)	0.538
*phi*D	4.05 (1.89–8.67)	<0.001*	3.44 (1.51–7.85)	0.003*	3.63 (0.98–13.43)	0.054	1.46 (0.34–6.31)	0.612

Predictor	AJCC Stage III or above (Stage3+)				Perineural invasion (PNI)			
	Univariable LR analysis		Multivariable LR analysis		Univariable LR analysis		Multivariable LR analysis	
	OR (95% CI)	*p*	AOR (95% CI)	*p*	OR (95% CI)	*p*	AOR (95% CI)	*p*
Age	1.06 (0.99–1.15)	0.097	1.10 (1.00–1.20)	0.049*	1.01 (0.95–1.07)	0.789	1.04 (0.96–1.14)	0.347
Prostate volume	0.97 (0.95–1.00)	0.056	0.98 (0.95–1.01)	0.139	0.98 (0.97–1.00)	0.067	0.98 (0.96–1.00)	0.064
# of positive cores ≥ 3	2.43 (1.02–5.79)	0.045*	2.13 (0.79–5.75)	0.134	2.43 (1.11–5.34)	0.027*	2.27 (0.90–5.76)	0.084
Total PSA	0.74 (0.40–1.36)	0.329	0.74 (0.33–1.68)	0.478	2.17 (0.70–6.70)	0.180	1.80 (0.73–4.40)	0.199
PSAD	1.17 (0.63–2.17)	0.615	1.02 (0.60–1.74)	0.950	2.03 (1.02–4.02)	0.043*	1.98 (0.93–4.19)	0.074
p2PSA	1.71 (0.92–3.18)	0.091	2.67 (1.13–6.32)	0.025*	1.05 (0.61–1.82)	0.859	1.10 (0.55–2.21)	0.785
p2PSAD	2.87 (1.46–5.67)	0.002*	2.91 (1.30–6.52)	0.009*	1.43 (0.83–2.47)	0.195	1.32 (0.69–2.51)	0.402
*phi*	1.83 (0.81–4.12)	0.145	1.45 (0.64–3.32)	0.374	2.95 (1.33–6.52)	0.008*	2.39 (0.97–5.91)	0.059
*phi*D	2.02 (1.12–3.67)	0.028*	1.71 (0.91–3.20)	0.093	2.05 (1.24–3.38)	0.005*	2.10 (1.17–3.80)	0.014*

A two‐tailed *p* value < 0.05 was considered statistically significant and is indicated by an asterisk (*).

**FIGURE 1:**
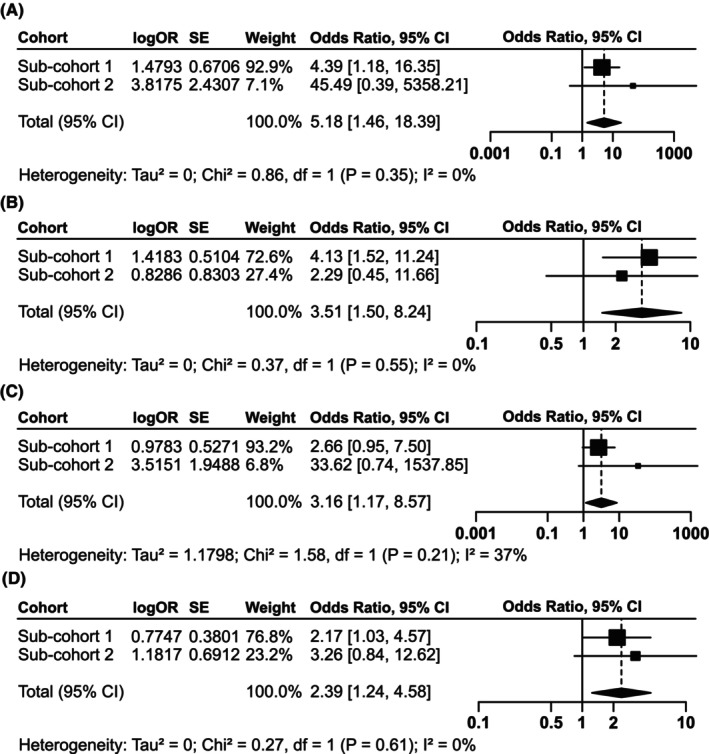
Meta‐analysis results for sub‐cohort 1 and 2 (tPSA <10 ng/mL subgroup). Meta‐analysis of sub‐cohort 1 and 2 examining the correlation between phi and pT3+ (A); phiD and pT3+ (B); phi and perineural invasion (C); phiD and perineural invasion (D). The above meta‐analyses were all conducted using a fixed‐effects model and employed the inverse‐variance weighting method.

**FIGURE 2:**
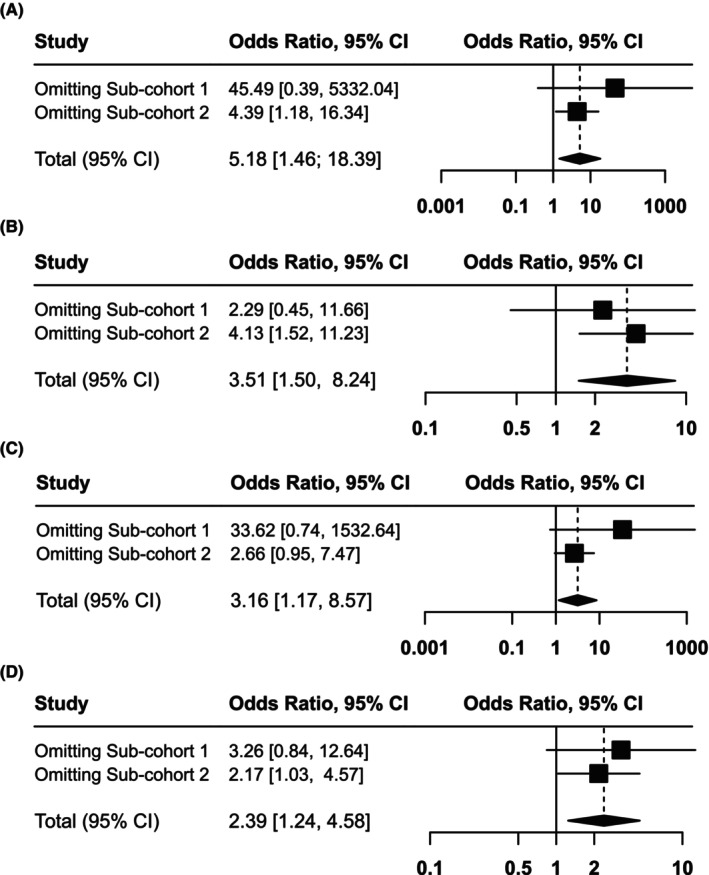
Sensitivity analysis results for sub‐cohort 1 and 2 (tPSA <10 ng/mL subgroup). Sensitivity analysis corresponding to the four meta‐analysis in Figure 1 respectively: phi and pT3+ (A); phiD and pT3+ (B); phi and perineural invasion (C); phiD and perineural invasion (D).

Additional analyses were performed separately in each of the two sub‐cohorts given the heterogeneity of the baseline characteristics (Table S2). To eliminate the effects of the heterogeneity on the study results, a meta‐analysis was conducted to obtain more accurate combined results (Figure 1) and a corresponding sensitivity analysis (Figure 2) across the two sub‐cohorts, mainly for *phi* and *phi*D predicting pT3+ and PNI because of their stable significance results and low *p*‐values shown above. No evidence of statistical heterogeneity was observed for two sub‐cohorts according to Figure 1 (all $I^{2}$ were <50%). The meta‐analysis results further affirmed the critical role of *phi* and *phi*D in predicting pT3+ (*phi*, pooled OR = 5.18, 95% CI: 1.46–18.39, *p* = 0.011; *phi*D, pooled OR = 3.51, 95% CI: 1.50–8.24, *p* = 0.004). What's more, *phi*D continues to exhibit its association with PNI (pooled OR = 2.39, 95% CI: 1.24–4.58, *p* = 0.009), and notably, *phi* demonstrates its predictive capability for PNI for the first time in the meta‐analysis (pooled OR = 3.16, 95% CI: 1.17–8.57, *p* = 0.024). The repeated sensitivity analysis in Figure 2 pointed out that our subgroup analysis results were primarily subjected to the sub‐cohort 1, with omitting the sub‐cohort 2 not influencing the final interpretation of the combined effect. This phenomenon might be partly attributed to a smaller sample size of the sub‐cohort 2 (*n* = 123) compared to the sub‐cohort 1 (*n* = 225).

Multiparametric MRI (mpMRI) has demonstrated high sensitivity and specificity in the diagnosis and staging of PCa.^18^ Based on the aforementioned research findings, we conducted a further analysis of sub‐cohort 3 (Table S3) to validate the predictive efficacy of *phi* and *phi*D for post‐RP adverse pathological features, specifically pT3+ and PNI (Table S4). Additionally, we aimed to explore whether *phi* can synergistically enhance the preoperative predictive performance of mpMRI for adverse pathological features following RP. Consistent with our previous findings, multivariable LR analysis indicated that both *phi* and *phi*D remained independent predictors of pT3+ (*phi*, AOR = 8.16, 95% CI: 1.51–44.10, *p* = 0.015; *phi*D, AOR = 2.36, 95% CI: 1.05–5.28, *p* = 0.037). *phi* was also found to be an independent predictor of PNI in this sub‐cohort (AOR = 6.36, 95% CI: 1.68–24.18, *p* = 0.004). And mpMRI demonstrated a comparable predictive value of pT3+ (AOR = 2.37, 95% CI: 1.02–5.47, *p* = 0.044). Notably, among patients with a false‐negative mpMRI result (*n* = 12), their median *phi* was 57.0, and 83.3% of these patients (10 out of 12) exhibited *phi* values exceeding the commonly used clinical threshold of 35.9, indicating the complementary roles between *phi*(D) and mpMRI.

## 4 DISCUSSION

In this observational, retrospective study, we explored the potential link between a range of *phi*(D) and adverse pathological features following radical prostatectomy (RP) in Chinese prostate cancer (PCa) patients. Our results demonstrated that both the *phi* and *phi*D are independent indicators for post‐RP specimens being classified as pathologic T stage pT3 or above (pT3+), as well as perineural invasion (PNI). Especially for pT3+ prediction, *phi* and *phi*D can synergise with mpMRI results. By incorporating these parameters, we can conduct a more comprehensive pre‐treatment risk assessment of PCa patients, which will facilitate the development of individualized radical treatment plans for those at elevated risk.

This study features the largest sample size thus far, focusing on the Chinese population from various regions. Furthermore, unlike some prior studies that relied solely on either biopsy results or postoperative pathological outcomes for GS determination,^19^ all participants in our study had access to reliable pathology results from both prostate biopsy and RP simultaneously. In addition, through conducting subgroup analyses and sensitivity analyses, and by including an additional sub‐cohort in the study to analyse the corresponding mpMRI data, we have validated the above conclusions. All these characteristics collectively enhance the validity and comprehensiveness of our conclusions.

In 2012, Guazzoni et al.^12^ were the first to investigate and demonstrate that %p2PSA (defined as p2PSA/fPSA) and the *phi* could predict final pathological outcomes such as pT3, GS ≥7, and GS upgrading. More recent studies^13^, ^14^, ^15^ have further strengthened the association of %p2PSA, *phi* and *phi*D with adverse PCa pathological features, primarily including pT3+ and GS ≥7. However, Fossati et al.^20^ conducted a decision curve analysis, revealing that models incorporating %p2PSA and *phi* did not significantly enhance net clinical decision‐making benefits. These dubious findings underscore the importance of further evaluating the predictive value of these biomarkers in clinical practice. Our study reaffirms the value of *phi* and *phi*D in the context of the Chinese population. Unfortunately, %p2PSA was not employed as a primary indicator in our research because, in China, p2PSA is primarily utilized as a component in calculating *phi* rather than in the form of %p2PSA.

It is evident that the adverse pathological features in these studies particularly related to pathologic T stage and GS. Our research, however, extends these considerations with a more comprehensive assessment approach. For instance, we have conducted a separate investigation into pT3b, which reflects seminal vesicle invasion (SVI), and included PNI as another evaluation criterion. Furthermore, we have added the AJCC prognostic stage grouping as another dimension of outcome assessment. This integration of serum tPSA level, TNM staging and Gleason grade group offers a higher degree of clinical relevance and prognostic value.^21^ Interestingly, as highlighted above, both p2PSA and p2PSAD showed a strong correlation with AJCC prognostic stage group III or higher. However, under the same analytical conditions, *phi* and *phi*D did not demonstrate a significant correlation based on the current study. This might be attributed to the effects arising during the AJCC grouping process. However, considering the inherent correlation between p2PSA and *phi*, our findings still align with and support the conclusions of previous researchers in this field.

mpMRI has become the standard of care for assessing the clinical staging of PCa and has proven useful in predicting adverse pathological features after RP.^22^ However, the predictive ability of mpMRI when combined with the *phi* remains underexplored. A limited number of studies have largely focused on specific subtypes of adverse pathological features such as extracapsular extension (ECE).^23^ Additionally, factors such as serum testosterone^24^ and the albumin/globulin ratio^25^ have also been associated with post‐RP adverse pathological features. These findings suggest that an ideal comprehensive predictive model for postoperative adverse pathological features should incorporate multifaceted parameters to enhance predictive accuracy. As mpMRI becomes increasingly accessible in various regions of China, the potential for combining serological and imaging data in larger population studies becomes more promising.

Our study has several limitations to be acknowledged. First, in the sub‐cohort 2, routine serum *phi* detection was not performed for patients with a tPSA value ≥10 ng/mL. Consequently, unlike the reported results by Eminaga et al.^26^ in which a specific cutoff value of 22.5 pg/mL for p2PSA to distinguish between organ‐confined and advanced PCa was suggested, we did not set a specific *phi* cutoff value but used the clinically established normal range for subsequent analyses. However, our findings remain significant for the population within the tPSA ‘grey zone’, which is precisely the demographic for whom the FDA approved the detection of p2PSA and *phi* in 2012. Second, the meta‐analysis indicated a low level of heterogeneity between the two constituent sub‐cohorts, suggesting the results were mainly driven by the sub‐cohort 1 due to its larger sample size. The differing results from the sub‐cohort 2 could be attributed to its relatively smaller sample size, which limits its statistical power. However, similar trends were observed based on the point estimate in sub‐cohort 2. Last but not least, despite the potential of mpMRI as a reliable tool for predicting pT3+, the mpMRI data referenced above was collected from a single medical centre and contains several missing values. Therefore, validating these findings through multi‐centre, large‐sample studies, and incorporating other multifaceted parameters to form a more ideal predictive model, may emerge as a valuable research direction in the future.

## 5 CONCLUSION

The detection of serum *phi* and *phi*D prior to RP in patients with PCa can aid in predicting the risk of adverse pathological features in postoperative specimens. This is particularly relevant for pathologic T stage pT3 or above, as well as perineural invasion. Our findings hold instructive value in forecasting the prognosis of post‐RP PCa patients. We anticipate that such predictive insights will be crucial in the holistic management of PCa patients, guiding the development of treatment plans meticulously tailored to each patient's circumstances.

## AUTHOR CONTRIBUTIONS

**Ruofan Shi:** Data curation (equal); formal analysis (equal); writing – original draft (equal). **Da Huang:** Data curation (equal); formal analysis (equal); writing – original draft (equal). **Jiaqi Yan:** Resources (equal). **Xiaohao Ruan:** Resources (equal). **Jingyi Huang:** Resources (equal). **Jiacheng Liu:** Resources (equal). **Jinlun Huang:** Resources (equal). **Yongle Zhan:** Resources (equal). **Chi Yao:** Resources (equal). **Tsun Tsun Stacia Chun:** Project administration (equal). **Brian Sze‐Ho Ho:** Project administration (equal). **Ada Tsui‐Lin Ng:** Project administration (equal). **Yi Gao:** Project administration (equal). **Danfeng Xu:** Conceptualization (equal); supervision (equal); writing – review and editing (equal). **Rong Na:** Conceptualization (equal); supervision (equal); writing – review and editing (equal).

## CONFLICT OF INTEREST STATEMENT

The reagent of tPSA, fPSA, p2PSA and *phi* tests in sub‐cohort 1 were provided by Beckman Coulter, Inc. The company did not involve in the study design, data analysis, results interpretation, manuscript writing, etc. in the current study. Otherwise, the authors report there are no competing interests to declare.

## ETHICS STATEMENT

The study protocol was approved by the Ethics Committee of Shanghai Ruijin Hospital (IRB No. 2020‐402, Mar 2 2021, version 2) and Institutional Review Board of the University of Hong Kong/Hospital Authority Hong Kong West Cluster (IRB No. UW16‐234). All subjects involved in the study gave their informed consent for inclusion before they participated in the study.

## Supporting information


Table S1: AJCC Prognostic Groups.

Table S2: Multivariable logistic regression results across sub‐cohort 1 and 2.

Table S3: Baseline clinical characteristics of sub‐cohort 3.

Table S4: Multivariable logistic regression results of sub‐cohort 3.

## Data Availability

The original data used or analysed in the study are included in the article and the supplementary material, further inquiries can be directed to the corresponding authors.
